# Combined metabolome and transcriptome profiling provides insights into dynamic molecular control of lemon (*Citrus Limon* L.) peel development

**DOI:** 10.1186/s12870-025-07674-5

**Published:** 2025-11-25

**Authors:** Hongming Liu, Chunrui Long, Shaohua Wang, Xiaomeng Fu, Jianmei Dong, Wei Yan, Meichao Dong, Minxian Duan, Birong Zhang, Hongxia Yang, Yuxia Du, Xianyan Zhou

**Affiliations:** Tropical and Subtropical Cash Crops Research Institute, Yunnan Academy of Agricultural Sciences, Baoshan, 678000 China

**Keywords:** *Citrus limon*, Peel development, Plant hormone, Metabolomics, Transcriptomics, Molecular pathways

## Abstract

**Background:**

Lemon peels are crucial quality traits and important sources of nutraceuticals. However, the regulation of peel development remains largely misunderstood. Here, we delved into the dynamic molecular mechanisms that control smooth lemon peel formation through transcriptomics and metabolomics analyses of developing peels at five different stages, including C1 (30 DAF, day after flowering), C2 (60 DAF), C3 (90 DAF), C4 (120 DAF), and C5 (150 DAF).

**Results:**

Our analyses revealed that peel development is stage-specifically regulated, with the transition from stage C2 to stage C3 being the most critical period. Notably, major metabolic adjustments and transcriptional switches occurred between C2 and C3. Particularly, phytohormones and their related genes, flavonoids, terpenoids, phenolic acids, vitamin C, and citric acid were down-regulated at stage C3. Differentially expressed genes between the transition from C2 to C3 were mainly enriched in hydrolase activity, nucleic acid binding transcription, transcription factor activity, sequence-specific DNA binding and pathways such as plant hormone signal transduction, starch and sucrose metabolism, MAPK signaling pathway-plant, plant-pathogen interaction, and circadian rhythm-plant biosynthesis. Plant hormone signaling was crucial for peel development process. Specifically, we found that crosstalks between jasmonic acid, abscisic acid, salicylic acid, gibberellic acid, and ethylene dynamically and coordinately regulate peel development process. ABC transporter family genes and green-degrading promoting genes, including protein SGR1 (*LOC18041639*), ervatamin-C (*LOC18051181*), and many ethylene-responsive transcription factors were significantly induced from C2. Furthermore, we screened out stage-specific most regulated genes.

**Conclusions:**

Our findings offer molecular understanding of peel formation process and fundamental resources for the functional dissection of its regulatory networks and for genomics-assisted control of peel shape and fruit quality in lemon.

**Supplementary Information:**

The online version contains supplementary material available at 10.1186/s12870-025-07674-5.

## Background

*Citrus limon* L. (lemon) is an important fruit in terms of nutrition, commerce, and medicine. belongs to the Rutaceae family [1]. Native to South Asia, lemon is cultivated in subtropical and tropical regions worldwide, with China being the top country producer [2, 3]. In China, lemon production has become a major industry, providing relatively higher profitability for farmers in the dry-hot valley region of Yunnan Province, the major lemon production zone [3]. The commercial value of lemon fruits depends mainly on appearance of the peel (colour and smoothness or roughness) and the composition of the pulp (juice percentage, soluble solids/acidity ratio, total phenolic content, etc.) [4]. However, many factors, including genetic diversity and the independent and autonomous molecular control of peel and pulp developmental processes as well as agronomic and environmental conditions, significantly affect the quality attributes and standard market indexes of lemon fruits [4]. Therefore, it is of the utmost importance to dissect and understand the molecular mechanisms that govern changes during peel and pulp formation.

The color of lemon fruit is primarily associated with the degreening process that occurs during the maturation, which involve the degradation of chlorophyll and the accumulation of carotenoids [5–8]. Both environmental conditions and endogenous factors, such as ethylene, ERF transcription factors, and the STAY-GREEN 1 protein, modulate the fruit degreening process [4, 5, 9]. For instance, Mitalo et al. discovered that ethylene and low temperature independently regulate genes involved in chlorophyll degradation, carotenoid metabolism, transcription factors, phytohormone biosynthesis and signaling, and photosystem proteins [9]. The formation of rough and smooth lemon skin is governed by differences in the regulation of metabolic processes, including asymmetric cell division, cell wall synthesis, signaling pathways (plant hormones and mitogen-activated protein kinases), ribosome pathways, and the biosynthesis of terpenoids, flavonoids, and phenylalanine [3]. In addition to influencing the fruit market valuethrough their appearance, lemon peels are exceptional reservoirs of bioactive phytochemicals with great potential for use in the food industry, packaging, edible films, bioremediation, and aquaculture [10]. Lemon peels are rich in phenolic acids (e.g., p-coumaric acid, ferulic acid, sinapic acid, chlorogenic acid, and caffeic acid), flavonoids (e.g., naringin, quercetin, and hesperidin), and terpenoids (e.g., d-limonene, γ-terpinene, and α-citral). These compounds are well documented for their pharmacological properties, including antioxidant, chemopreventive, anti-inflammatory, antimicrobial, cardioprotective, antifungal, and anticancer bioactivities [10–12]. Despite their commercial and industrial importance, the molecular regulation of peel development has not yet been investigated. Elucidating the dynamic molecular mechanisms involved in peel development and identifying key regulatory elements will help control the appearance of lemon fruits and improve fruit quality.

The present study addresses this research gap. We aimed to provide comprehensive molecular insights into the developmental process of lemon peels and fundamental resources for further functional studies toward molecular-assisted control of peel appearance and quality. Through an in-depth analysis of changes in developing peels’ metabolite profiles and transcriptome patterns at five different stages after flowering, we comprehensively explored the dynamic molecular regulatory mechanisms controlling lemon peel formation. Our results will prompt further research on the molecular control of lemon peel appearance and quality, as well as the overall quality of lemon fruits.

## Results and discussion

### Dynamic metabolite profile changes during lemon peel development

**Fig. 1:**
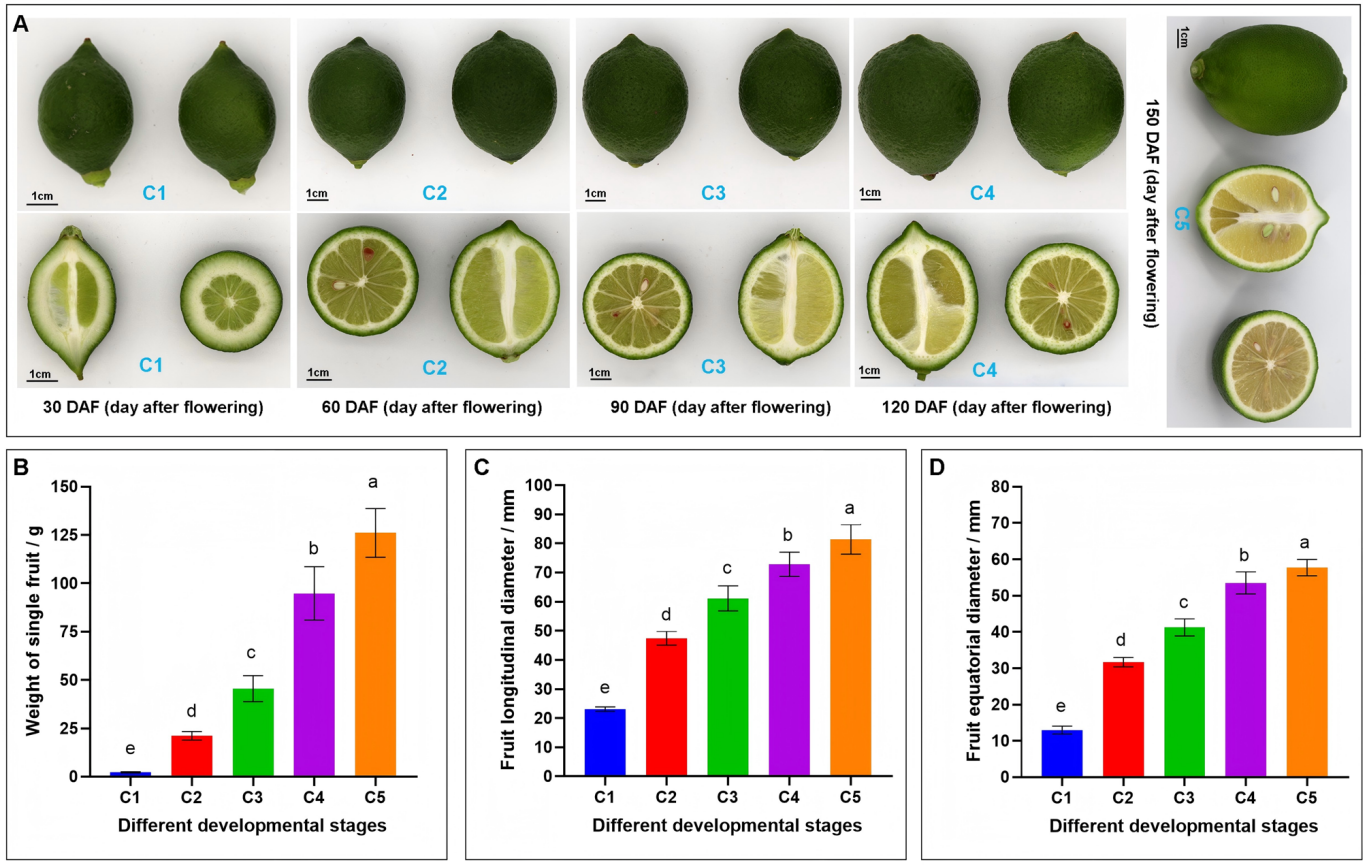
Morphological characteristics of developing lemon peels at five different stages. **A** Image of whole and segmented lemon fruits at the five stages. **B** Weight of a single fruit at the five stages. **C** Longitudinal diameter of fruits at the five stages. **D** Equatorial diameter of fruits at the five stages. C1, C2, C3, C4, and C5 indicate developing lemon fruits at 30 DAF (day after flowering), 60 DAF, 90 DAF, 120 DAF, and 150 DAF, respectively

To explore metabolome shifts and the dynamic metabolic regulation occurring during smooth lemon peel development, we took advantage of our previously released global metabolome data of developing peels of ‘Yunning No.1’ lemon at five stages, including C1 (30 DAF), C2 (60 DAF), C3 (90 DAF), C4 (120 DAF), and C5 (150 DAF) [3]. The phenotypes of the fruits at these stages are presented in Fig. 1A. The average weight, average longitudinal diameter, and average equatorial diameter of a single fruit at stage C5 are 126.09 g, 81.41 mm, and 57.73 mm, respectively (Fig. 1B-D). A total of 1,682 metabolites were identified at the positive and negative ion modes, including lipids, ketones, aldehydes and esters, terpenoids, phenolic acids, sugars, organic acids, flavonoids, etc. (Table S1). It is known that lemon peels are rich sources of important phytochemicals for diverse industrial applications [10, 13]. This data represents an essential resource for an in-depth overview of the metabolite profile of lemon peels and may guide future targeted molecular studies. However, targeted metabolomics analyses coupled with bioactivity assessments are required to accurately capture the phytochemical profile of peels and understand their various functional abilities.

**Fig. 2:**
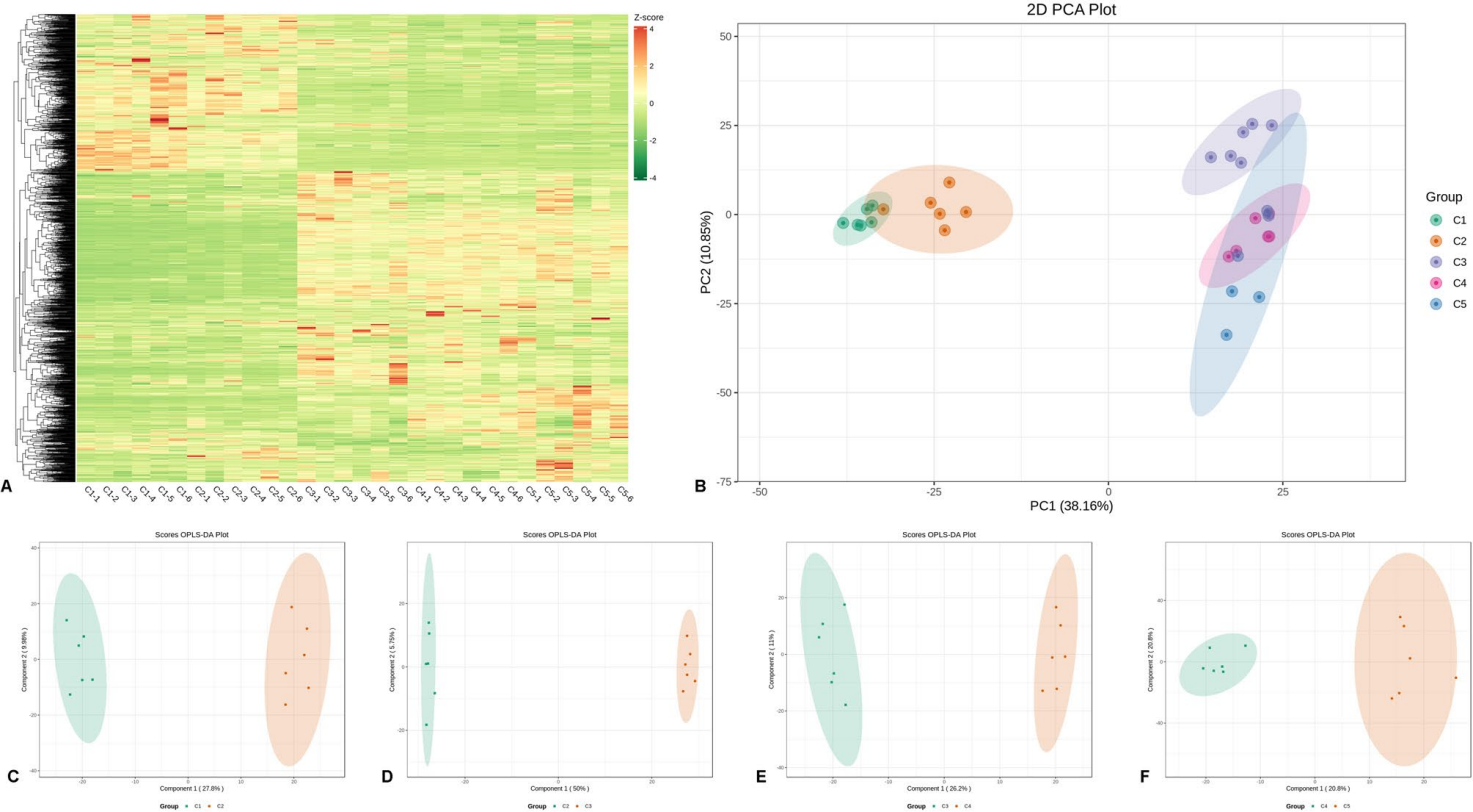
Metabolite profiles of developing lemon peels at five different stages. **A** Hierarchical clustering analysis. **B** Principal component analysis. **C**-**F** OPLS-DA plots of pairwise comparison between C1.vs.C2, C2.vs.C3, C3.vs.C4, and C4.vs.C5, respectively. C1, C2, C3, C4, and C5 indicate developing lemon peels at 30 DAF (day after flowering), 60 DAF, 90 DAF, 120 DAF, and 150 DAF, respectively

Peels at stages C1 and C2 exhibited considerable differences in metabolite profiles compared to those at C3, C4, and C5 (Fig. 2A, B). Notably, peels at stages C1 and C2 clustered closely on the PCA plot and exhibited similar patterns of metabolite accumulation (Fig. 1A, B). Samilarly, peels at stages C3, C4, and C5 also clustered together and could be distinguished from the C1 and C2 group by PC1 of 38.16% (Fig. 2A, B). These results suggest that important metabolic switches occurred in developing peels between 60 (C2) and 90 (C3) DAF. To verify the observed metabolome profile changes, we conducted pairwise OPLS-DA analyses. The OPLS-DA models showed strong goodness of fit and reliability, with an R^2^Y ˃ 0.96 and Q^2^Y ˃ 0.78 (Fig. S1). The score plots of OPLS-DA supported the important metabolic switches in developing peels between 60 DAF and 90 DAF (Fig. 2D). The score plots of OPLS-DA for the comparisons between C1.vs.C2, C3.vs.C4, and C4.vs.C5 confirm that no major metabolome shifts occurred during the transitions between these stages (Fig. 1C, E, F). Collectively, these results suggest that the lemon peel developmental process could be divided into three main stages: (1) the period from 0 to 60 DAF; (2) the transition from 60 to 90 DAF; and (3) the period from 90 DAF to ripening. The molecular changes between the transition from 60 to 90 DAF might be critical for high-quality fruit formation in terms of appearance and nutritional composition. Understanding mechanisms involved in this key transitional regulation is essential for the molecular-assisted control of lemon fruit quality.

### Differential metabolites (DMs) confirm important metabolic adjustment in developing peels between 60 and 90 DAF

**Fig. 3:**
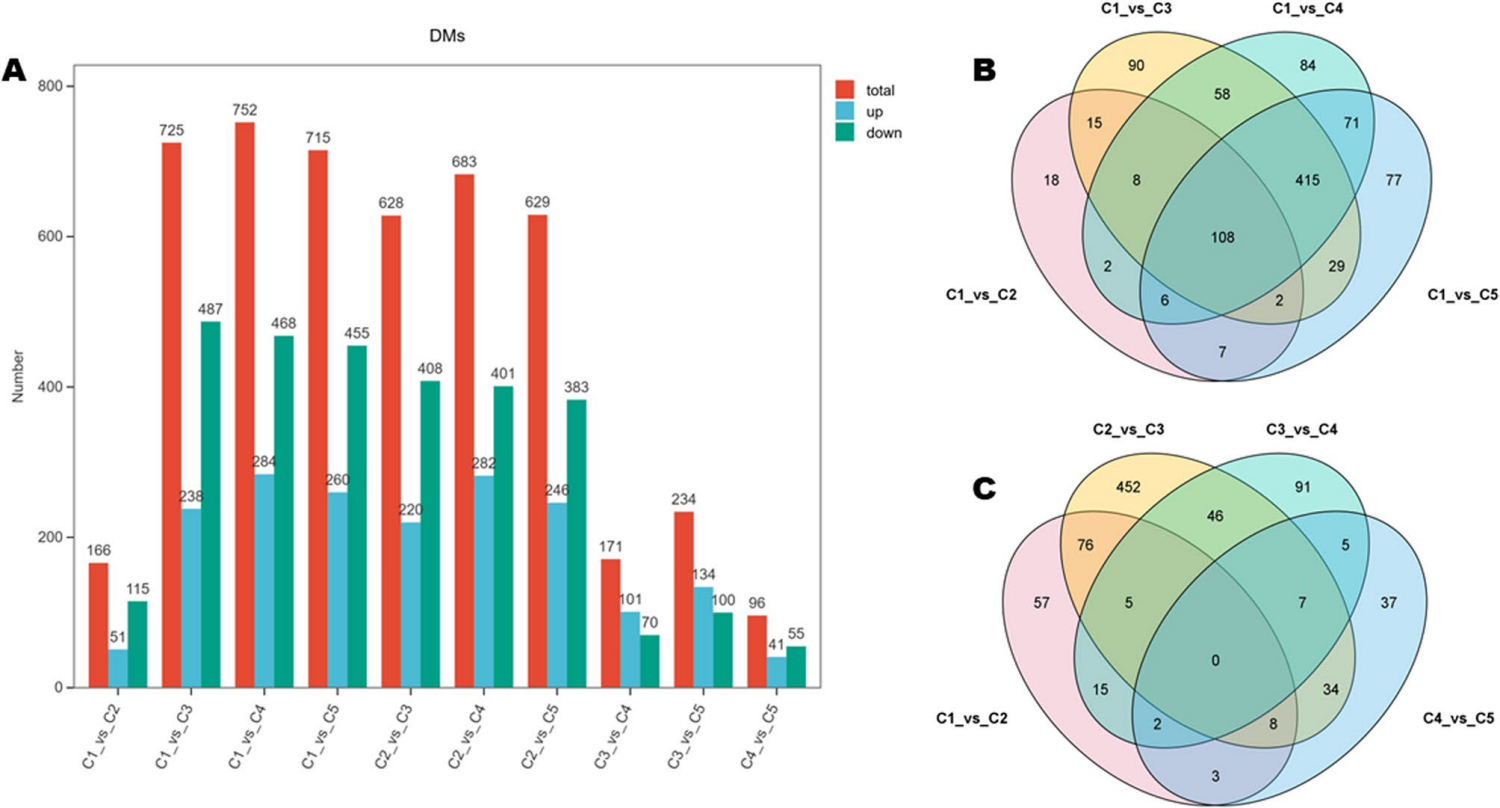
Differential metabolites (DMs). **A** Number of DMs in all pairwise comparisons. **B** Venn diagram of common DMs between C1.vs.C2, C1.vs.C3, C1.vs.C4, and C1.vs.C5. **C** Venn diagram of overlapped DMs between C1.vs.C2, C2.vs.C3, C3.vs.C4, and C4.vs.C5. C1, C2, C3, C4, and C5 indicate developing lemon peels at 30 DAF (day after flowering), 60 DAF, 90 DAF, 120 DAF, and 150 DAF, respectively

DMs offer the opportunity to identify differentially regulated pathways (DPs) and enable an overview of changes in metabolic processes. We uncovered a total of 96 (C4.vs.C5) to 752 (C1.vs.C4) DMs in pairwise comparison between groups (Fig. 3A). Notably, we detected 166 (including 51 up-regulated in C2), 628 (including 220 up-regulated in C3), 171 (including 101 up-regulated in C4), and 96 (including 41 up-regulated in C5) between C1.vs.C2, C2.vs.C3, C3.vs.C4, and C4.vs.C5, respectively, with no common DMs (Fig. 3A, C). The number of DMs between C2.vs.C3 was higher compared to C1.vs.C2, C3.vs.C4, and C4.vs.C5. These results indicate a stage-specific metabolome regulation during peel development in lemon, particularly between C2 and C3 stages. The higher number of down-regulated DMs between C2.vs.C3 suggests major transcriptional control of metabolic processes during this transition. When we considered C1 as the control group, we identified 108 overlapped DMs (Fig. 3B), with the highest numbers of DMs detected between C1.vs.C3 (725 DMs), C1.vs.C4 (752 DMs), and C1.vs.C5 (715 DMs) (Fig. 2A). The similar number of DMs between C1.vs.C3, C1.vs.C4, and C1.vs.C5 provides support to the closeness of the metabolite profiles of developing peels at stages C3, C4, and C5. We carried out KEGG analysis of DMs between C1.vs.C2, C2.vs.C3, C3.vs.C4, and C4.vs.C5 to unveil dynamic DPs during lemon peel development. DMs between C1.vs.C2 were primarily assigned to biosynthesis of secondary metabolites and flavonoid biosynthesis (Fig. S2A). Meanwhile, DMs between C2.vs.C3 were mostly involved in biosynthesis of secondary metabolites, flavonoid biosynthesis, biosynthesis of cofactors, biosynthesis of amino acids, nucleotide metabolism, ABC transporters, and isoquinoline alkaloid biosynthesis (Fig. S2B). Biosynthesis of secondary metabolites, biosynthesis of amino acids, biosynthesis of cofactors, carbon metabolism, and tryptophan metabolism were the major DPs between C3.vs.C4 (Fig. S3A). The major DPs between C4.vs.C5 were biosynthesis of secondary metabolites and ABC transporters (Fig. S3B). These results show that the dynamic regulation of secondary metabolite biosynthesis is crucial for normal peel formation. More metabolic pathways were adjusted between stages C2 and C3, further confirming important metabolic switches during this transition. These findings suggest that intensive cell division and expansion processes may occur between 60 and 90 DAF to constitute peel tissues. It is well known that cell proliferation necessitates extensive metabolic adjustment to allow cells to acquire sufficient nutrients (e.g., glucose, amino acids, lipids, nucleotides, etc.) that are essential to support cell growth and antioxidant capacity [14, 15].

**Fig. 4:**
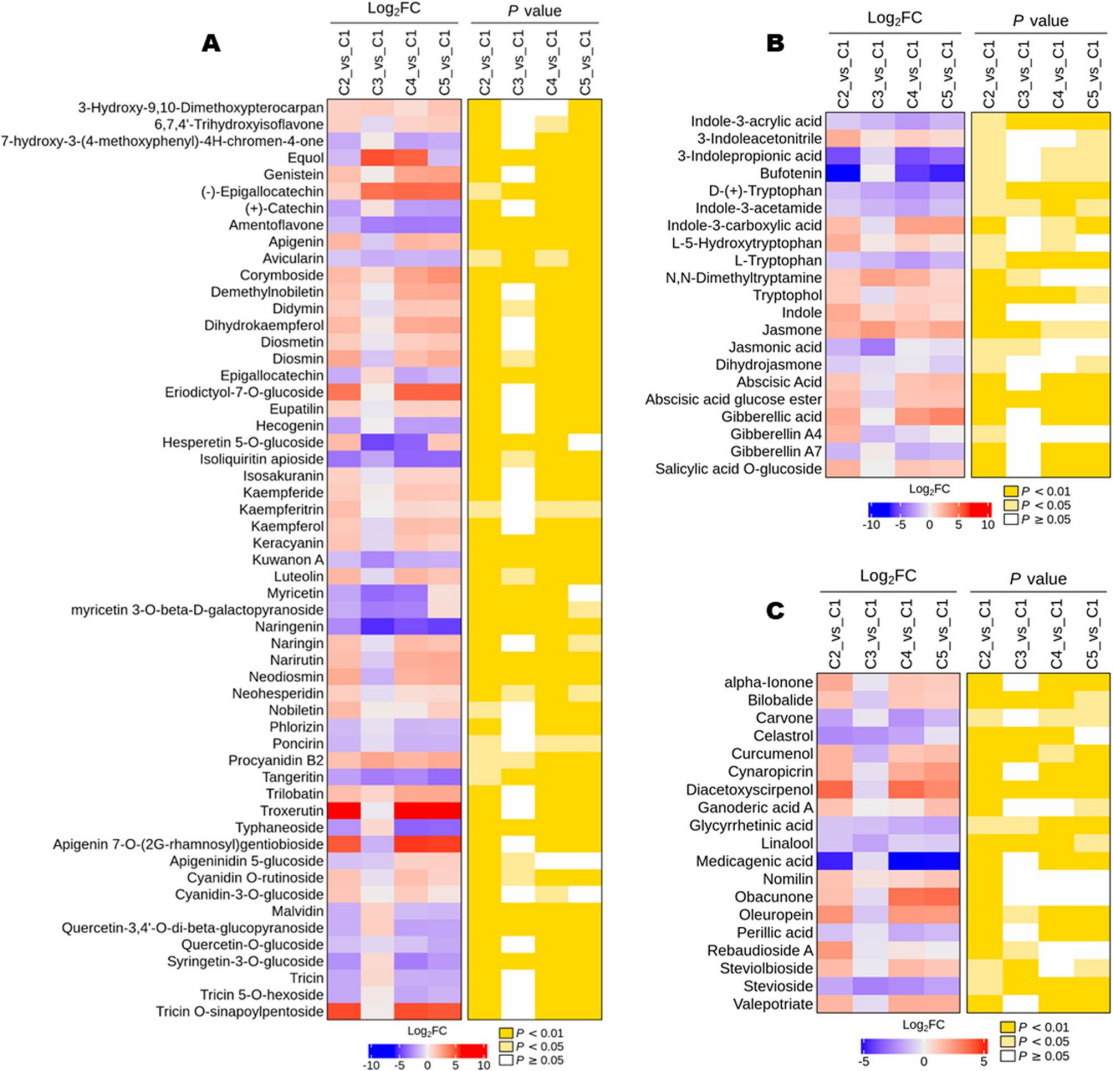
Accumulation patterns of key DMs in developing lemon peels.** A** Flavonoids; **B** Indole compounds and phytohormones; **C** Terpenoids. C1, C2, C3, C4, and C5 indicate developing lemon peels at 30 DAF (day after flowering), 60 DAF, 90 DAF, 120 DAF, and 150 DAF, respectively

To get insight into this metabolic adjustment between C2 and C3, we examined the dynamic changes in the accumulation patterns of key peel quality traits, including differential flavonoids, terpenoids, phenolic acids, and carbohydrates (Fig. 4 and S5). Most differential flavonoids, terpenoids, phenolic acids, and vitamin C showed significantly low accumulation patterns in peels at stage C3 compared to others (Fig. 4A, C and S5). Most differential carbohydrates and citric acid exhibited similar low accumulation patterns in peels at stage C3; however, the differences were not significant (Fig. S5). Catechin derivatives, procyanidin B2, malvidin, tricin, syringetin-3-*O*-glucoside 4-coumaric acid, benzamide, and vanillic acid had the highest accumulation in peels at stage C3 (Fig. 4A and S5). These findings suggest that the major metabolic adjustments between C2 and C3 are related to resource mobilization to sustain cell proliferation and the formation of peel tissue structural components. (Fig. 4A, C and S5). Plant hormones, including auxin, gibberellic acid (GA), jasmonic acid (JA), salicylic acid (SA), and abscisic acid (ABA) regulate the complex processes of ovary-to-fruit transformation and fruit formation and therefore play a significant role in improving the quality and biochemical composition of fruits [16, 17]. Auxin modulates early fruit development and fruit size through the regulation of cell division and expansion [16, 18]. Indole compounds, precursors of auxin, stimulate fruit formation and activate the defense system of plants against biotic and abiotic factors [19]. Cross-talk of GA with other hormones, such as ABA and JA, is critical for fruit set, development, and ripening. GA promotes cells’ expansion, increasing fruit weight without any loss of quality traits, and controls chlorophyll and carotenoid metabolism to prevent fruit ripening [20]. ABA modulates fruit growth, ripening, and senescence [21, 22]. JA is a major regulator of fruit development through crosstalk with other phytohormones to balance development processes and environmental response mechanisms [17, 23]. SA and its derivatives play important roles in flowering and fruit ripening, improving fruit quality and content of antioxidant compounds [24]. The contents of ABA, JA, GA, salicylic acid-*O*-glucoside, and most indole compounds in peels at stage C3 were the lowest (Fig. 4B). These results further confirm the transition from C2 to C3 as the pivotal stage of peel development. Moreover, they suggest a coordinated reprogramming of metabolic processes in developing peels during the transition from C2 to C3. ABA, JA, GA, and salicylic acid-*O*-glucoside showed upregulated contents from stage C4 (Fig. 4B), indicating crosstalk between them might be essential for peel ripening and color formation. Further studies targeting the key developmental period (between C2 and C3) and investigating the specific role of these phytohormones are needed to improve understanding of the regulation of peel formation and lemon quality.

### Dynamic transcriptome changes during lemon Peel development

**Fig. 5:**
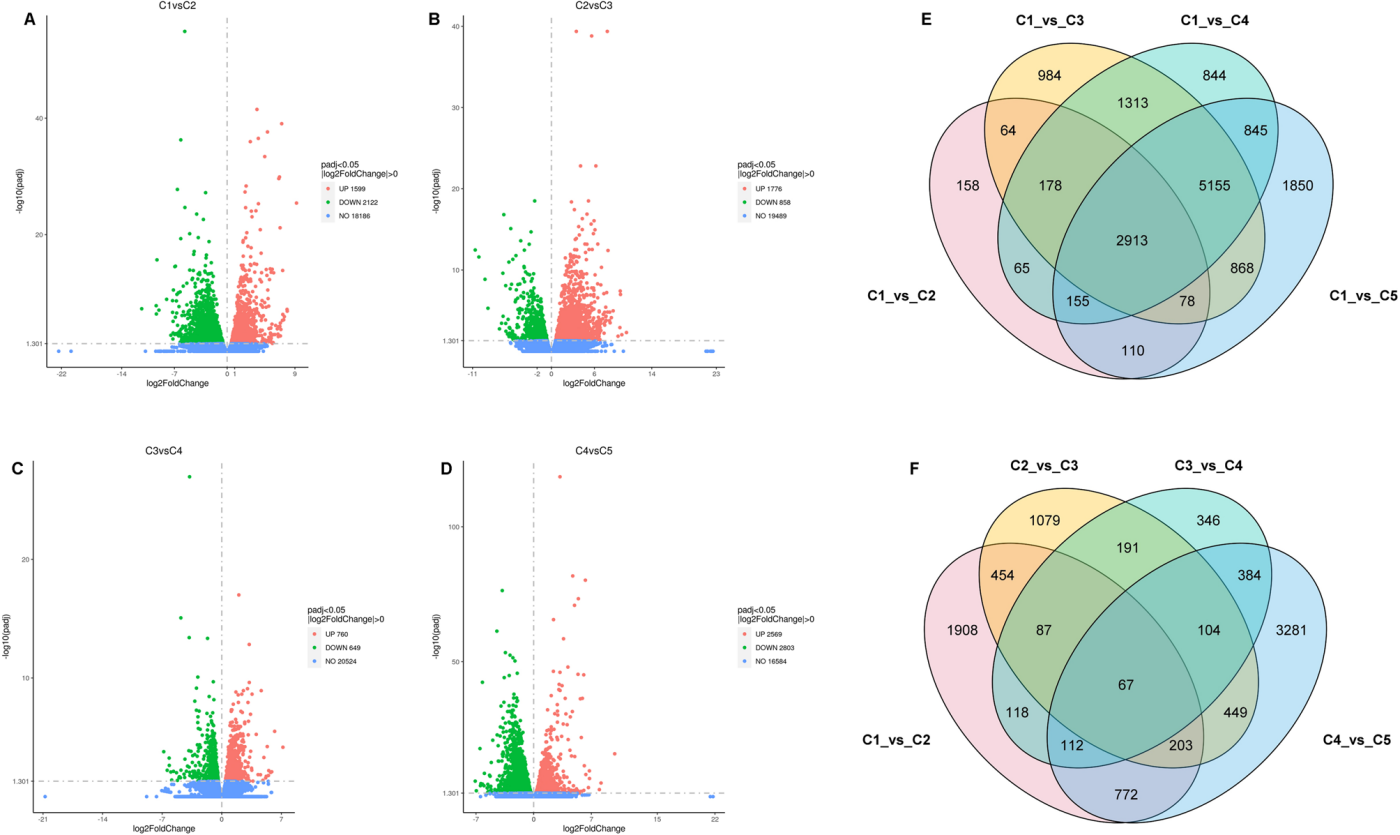
Transcriptome profiles of developing lemon peels at five different stages. Volcano plot of DEGs between C1.vs.C2 **A**, C2.vs.C3 **B**, C3.vs.C4 **C**, and C4.vs.C5 **D**.** E** Venn diagram of common DMs between C1.vs.C2, C1.vs.C3, C1.vs.C4, and C1.vs.C5. **F** Venn diagram of overlapped DMs between C1.vs.C2, C2.vs.C3, C3.vs.C4, and C4.vs.C5. C1, C2, C3, C4, and C5 indicate developing lemon peels at 30 DAF (day after flowering), 60 DAF, 90 DAF, 120 DAF, and 150 DAF, respectively

To gain more molecular insights into the lemon peel development process, we analyzed our previously released transcriptome data of the five stages [3]. The summary of the high-quality RNA-seq data is presented in Table S2. Consistent with the metabolite profiles, the highest numbers of DEGs (differentially expressed genes) were detected between C1.vs.C3 (11,553 DEGs), C1.vs.C4 (11,668 DEGs), and C1.vs.C5 (11,974 DEGs) (Fig. S5). These results align with the metabolome profiling and suggest similar metabolic regulation of peel formation from 90 DAF till fruit ripening. There were 3,721 (including 1,599 up-regulated in C1), 2,634 (including 1,776 up-regulated in C2), 1,409 (including 760 up-regulated in C3), and 5,372 (including 2,569 up-regulated in C4) DEGs between C1.vs.C2, C2.vs.C3, C3.vs.C4, and C4.vs.C5, respectively (Fig. 5A-D). Only 67 DEGs were common between these four pairwise comparisons (Fig. 5F), which support that different transcriptional regulation processes occurred during transitions between the five stages. When we considered C1 as the control group, we identified 2,913 overlapped DEGs (Fig. 5E). Among the 2,634 DEGs between C2.vs.C3, 67.42% (1,776 DEGs) were down-regulated at the stage C3. This result aligns with the significant decrease in the accumulation of key metabolites in peels at this stage, and supports important molecular switches during the transition from stage C2 to stage C3.

### Dynamic molecular mechanisms during lemon Peel development

To reveal the key molecular regulatory mechanisms of peel development in lemon, we performed GO and KEGG enrichment analyses of DEGs. Functional annotation of DEGs to the GO and enrichment analysis revealed that DEGs between C1.vs.C2 were primarily associated with oxidoreductase activity, transferase activity, iron ion binding, single organism metabolic process, ATP generation from ADP, glycolytic process, and nucleoside diphosphate phosphorylation (Fig. S6A), which are indicative of intensive cell division and expansion processes. DEGs between C2.vs.C3 were mostly involved in hydrolase activity, nucleic acid binding transcription, transcription factor activity, sequence-specific DNA binding, carbohydrate metabolic process, single-organism carbohydrate metabolic process, and cellulose metabolic process (Fig. S6B). These results confirm that key transcriptional regulation and metabolic reprogramming occur in developing lemon peels between 60 DAF and 90 DAF. Meanwhile, transferase activity, hydrolase activity, lyase activity, and carbohydrate metabolic process were the major GO terms between C3.vs.C4 (Fig. S6C). The major GO terms between C4.vs.C5 were hydrolase activity, pyrophosphatase activity, cytoskeletal protein binding, carbohydrate metabolic process, intracellular signal transduction, microtubule-based process, motor activity, and tubulin binding (Fig. S6D).

**Fig. 6:**
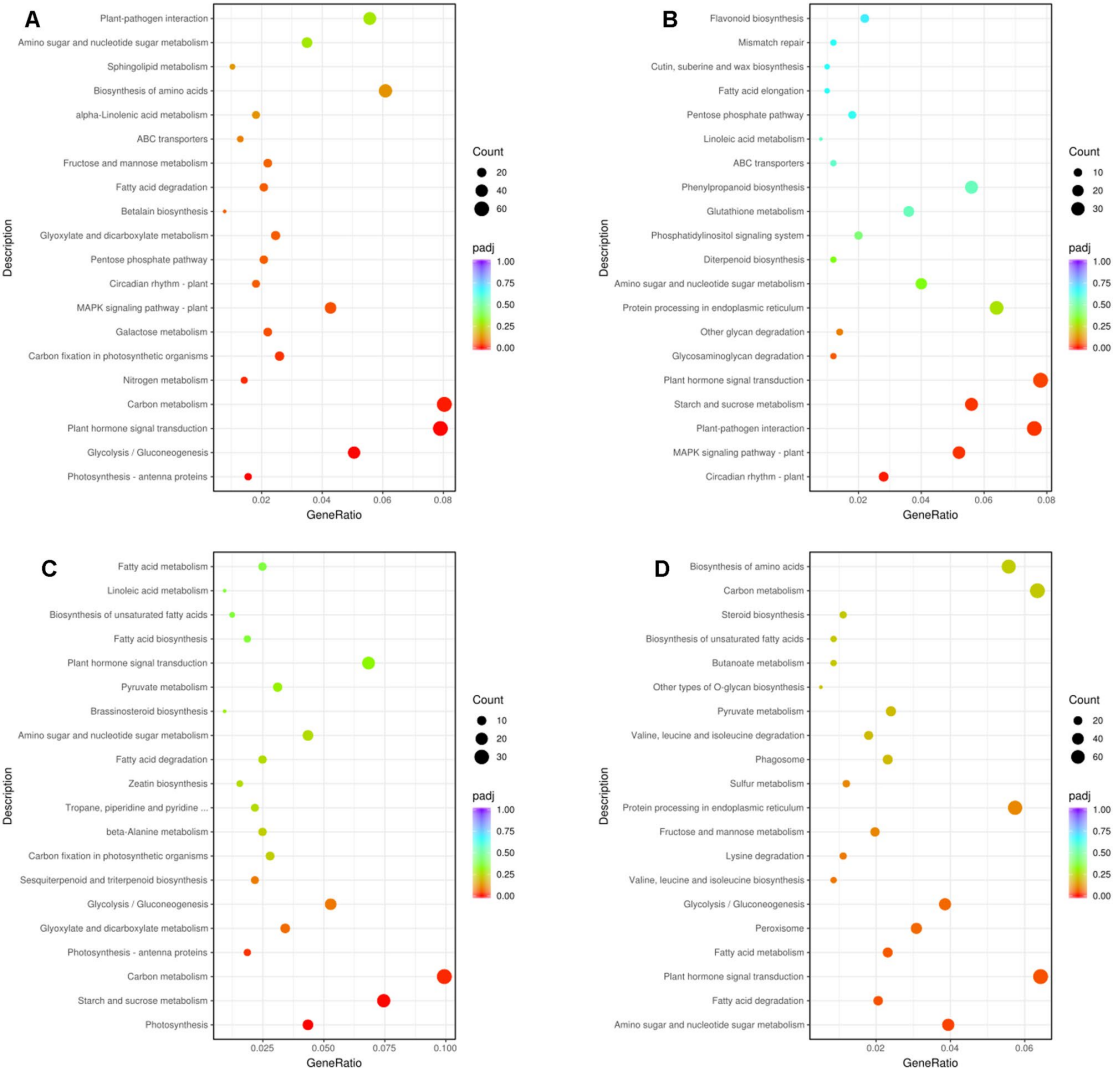
Functional annotation of DEGs. KEGG annotation and enrichment analysis of DEGs between C1.vs.C2 **A**, C2.vs.C3 **B**, C3.vs.C4 **C**, and C4.vs.C5 **D**. C1, C2, C3, C4, and C5 indicate developing lemon peels at 30 DAF (day after flowering), 60 DAF, 90 DAF, 120 DAF, and 150 DAF, respectively

Functional annotation of DEGs to the KEGG pathways and enrichment analysis revealed that DEGs between C1.vs.C2 were primarily involved in plant hormone signal transduction, carbon metabolism, glycolysis/gluconeogenesis, galactose metabolism, MAPK signaling pathway-plant, and carbon fixation in photosynthetic organisms (Fig. 6A), indicating a mobilization of metabolic resources to sustain the intensive cell division and expansion processes. DEGs between C2.vs.C3 were mostly involved in plant hormone signal transduction, starch and sucrose metabolism, MAPK signaling pathway-plant, plant-pathogen interaction, and circadian rhythm-plant (Fig. 6B). Meanwhile, photosynthesis, starch and sucrose metabolism, carbon metabolism, glyoxylate and dicarboxylate metabolism, and glycolysis/gluconeogenesis were the major DPs between C3.vs.C4 (Fig. 6C). The major DPs between C4.vs.C5 were plant hormone signal transduction, amino sugar and nucleotide sugar metabolism, fatty acid metabolism, peroxisome, glycolysis/gluconeogenesis, and protein processing in endoplasmic reticulum (Fig. 6D). These findings enlighten the importance of signaling pathways in coordinating smooth lemon peel formation, notably during early stages. Previous studies have highlighted the crucial roles of phytohormones and TFs in regulating fruit initiation, development, and ripening [5, 25, 26].

## Key DEGs modulating peel developmental process in lemon

**Fig. 7:**
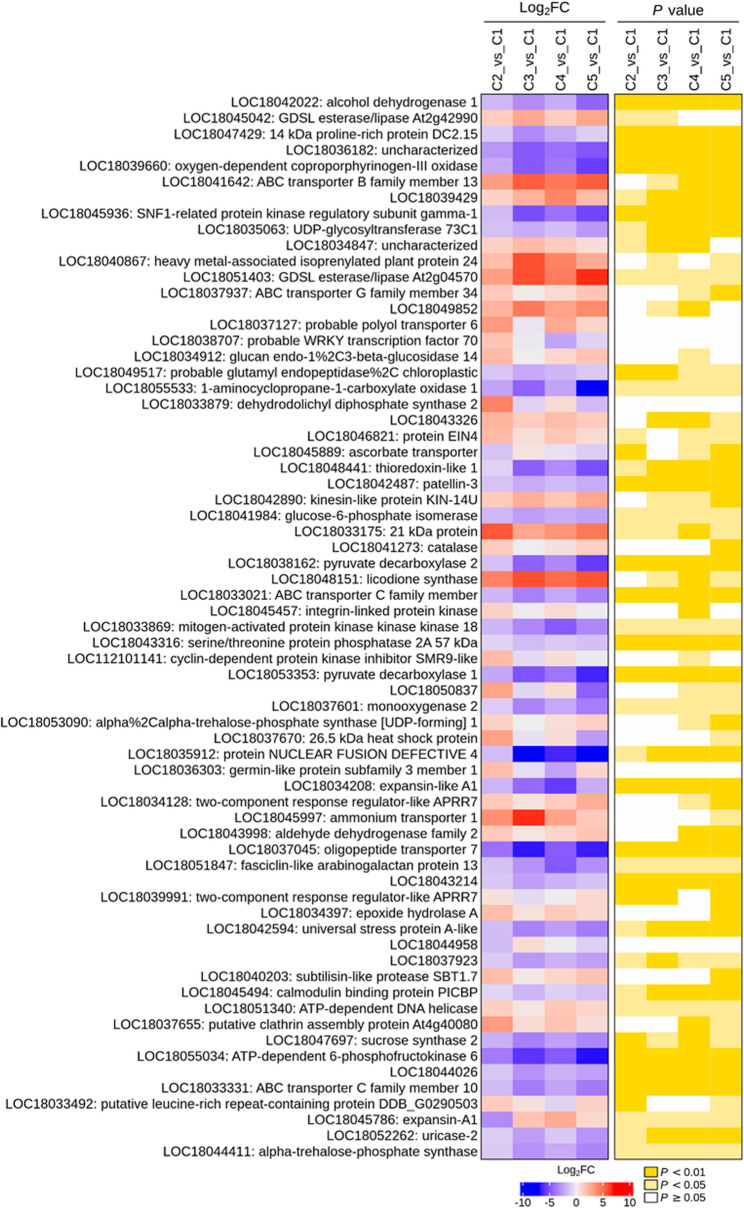
Expression patterns of common 67 DEGs between C1.vs.C2, C2.vs.C3, C3.vs.C4, and C4.vs.C5. C1, C2, C3, C4, and C5 indicate developing lemon peels at 30 DAF (day after flowering), 60 DAF, 90 DAF, 120 DAF, and 150 DAF, respectively

To identify key DEGs involved in the control of lemon peel development and quality, we first examined the expression patterns of the 67 common DEGs between C1.vs.C2, C2.vs.C3, C3.vs.C4, and C4.vs.C5. As shown in Fig. 7, most of these genes were down-regulated at stage C3. The up-regulated DEGs at stage C3 include ascorbate transporter (*LOC18045889*), heavy metal-associated isoprenylated plant protein 24 (*LOC18040867*), ammonium transporter 1 (*LOC18045997*), expansin-A1 (*LOC18045786*), etc. (Fig. 7). These genes remained highly expressed up to stage C5, indicating the importance of transporters during lemon fruit development, particularly in mediating metabolic flux in peels during late developmental stages. For instance, ammonium transporters mediate ammonium uptake, which plays crucial functions in plant growth, development, fruit formation and fruit quality [27]. Lemon peels are a rich source of ascorbate (vitamin C) [10, 13]. The induction of ascorbate transporter infers that vitamin C is not only a quality trait in lemon but also a key regulator of fruit developmental processes. Indeed, previous studies revealed that vitamin C plays pivotal roles in various plant physiological processes, including cell division and differentiation, and photosynthesis, making it a critical molecule for plant growth, development, and environmental resilience [28, 29]. In contrast, protein nuclear fusion defective 4 (*LOC18035912*) and dihydrodolichyl diphosphate synthase 2 (*LOC18033879*) were the most down-regulated genes at stage C3 (Fig. 7). The transcription factor WRKY70 (*LOC18038707*), germin-like protein subfamily 3 member 1 (*LOC18036303*), and putative leucine-rich repeat-containing protein DDB_G0290503 (*LOC18033492*) were induced at stages C2 and C5 (Fig. 7). WRKY TFs regulate carbohydrate metabolism and tolerance to pathogens during fruit development in *Citrus sinensis* [30, 31]. ABC transporters were mainly induced along the peel development (Fig. 7), showing that they are critical for the coordinated growth and development of lemon fruits. ABC transporters transport complex organic materials against concentration gradients, providing key complex building blocks essential for the development of specialized plant cells [32]. In *Citrus medica*, *CmABCB19* and *CmABCC10* mediate normal fruit development by controlling auxin and flavonoid synthesis and transport [33].

**Fig. 8:**
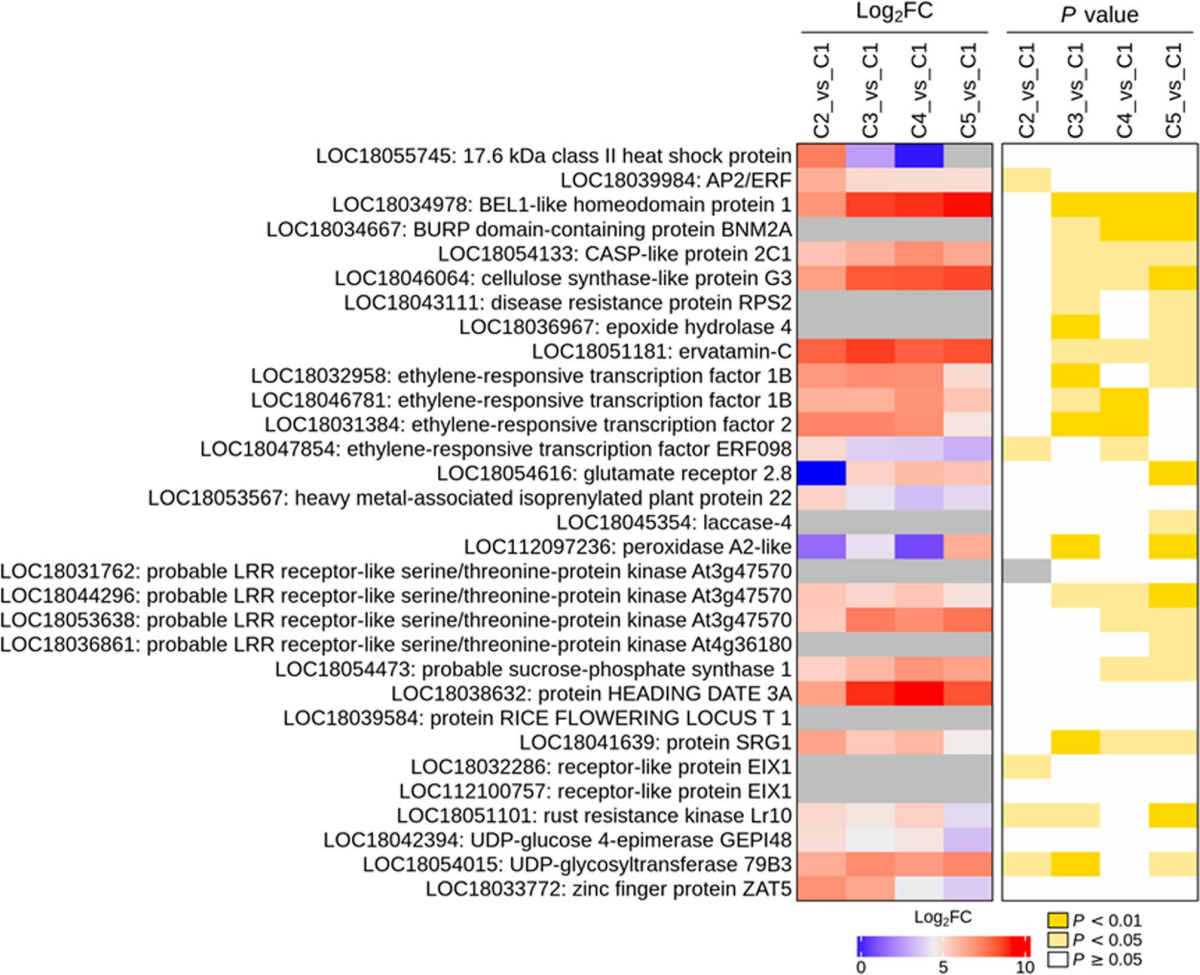
Expression patterns of most differentially regulated DEGs during peel formation, with induced expression during late stages. C1, C2, C3, C4, and C5 indicate developing lemon peels at 30 DAF (day after flowering), 60 DAF, 90 DAF, 120 DAF, and 150 DAF, respectively

Finally, we screened out the most significant DEGs (|Log2FC| ˃ 5) during lemon peel development (Table S3). In total, 379 DEGs were filtered out. Phytohormones are crucial for plant developmental processes. Three probable indole-3-acetic acid-amido synthetase GH3.1 (*LOC18033692*,* LOC18053209*, and *LOC18035901*) and one probable indole-3-pyruvate monooxygenase YUCCA4 (*LOC18055290*) were over 5.6-fold down-regulated at stage C2 (Table S3), indicating they were significantly induced at stage C1 to stimulate cell division and expansion. Auxin-responsive protein SAUR21 (*LOC18035415*) was over 5.2-fold down-regulated at stage C4 (Table S3). DELLA protein RGL1 (*LOC18031396*) was over 5.4-fold down-regulated at stage C2 (Table S3), implying that it negatively regulates fruit development. As support, studies in *Arabidopsis*revealed that DELLA proteins repress fruit development [20]. Gibberellin-regulated protein 14 (*LOC18038199*) and 4 (*LOC18039760*) were over 6.2-fold down-regulated at stage C3, then up-regulated from stage C4 (Table S3). The expression patterns of auxin and gibberellin-related genes are consistent with dynamic changes in the levels of phytohormones in developing peels. Previous studies highlighted the crucial roles of phytohormones and TFs in regulating fruit ripening [4, 5, 25, 26]. Gibberellins prevent citrus fruit cracking by stimulating cell division and expansion, leading to thicker peel formation [26]. Therefore, the up-regulation of *LOC18038199* and *LOC18039760* from stage C4 suggests that they may promote peel thickness to prevent fruit cracking. At stage C3, F-box protein *At1g30790* (*LOC18044672*) was significantly up-regulated, while F-box protein *At2g26160* (*LOC18054322*) and F-box protein PP2-B15%2 C transcript variant X2 (*LOC18039004*) were down-regulated (Table S3). Li et al. found that the F-box protein *STERILE APETALA*1 (*SlSAP1)* and *SlSAP2* redundantly regulate leaf and fruit size in tomato by modulating the stability of *SlKIX8* and *SlKIX9* [34]. Fig. 8 presents the most significantly induced DEGs from stage C2. Protein SGR1 (*LOC18041639*), glutamate receptor (*LOC18054616*), ervatamin-C (*LOC18051181*), and many ethylene responsive transcription factors and disease resistant-related genes were induced from stage C2 (Table S3, Fig. 8). Ervatamin-C genes are stable papain-like cysteine proteases that play important roles in plant development, leaf senescence, seed germination, defense mechanisms, etc [35]. The induction of *LOC18051181* from stage C2 indicates that it may play crucial regulatory functions during peel development. Chlorophyll degradation coupled with carotenoid accumulation is a critical process of fruit maturation in numerous horticultural plants [5–8]. The balance between chlorophyll, carotenoid, and flavonoid contents modulates fruit color and quality [5–8]. Ethylene-responsive transcription factors and STAY-GREEN genes positively regulate chlorophyll degradation and fruit degreening [5, 6]. JA and ABA modulate the expression of genes related to ethylene biosynthesis and regulate ethylene biosynthesis and signaling during fruit ripening [17, 21, 23]. The effects of ABA and ethylene on fruit ripening are synergistic [21]. The metabolomics analysis disclosed high levels of JA, ABA, SA, and GA in developing peels from stage C4 (Fig. 4B). Collectively, these results show that lemon peel development is dynamically coordinately regulated by crosstalks between phytohormones and TFs, principally JA, ABA, SA, GA, and ethylene. Assessing the molecular functions of identified degreening-promoting genes will enhance our understanding of the regulatory mechanisms controlling the color of citrus fruits. Notably, functional characterization of phytohormone-related genes and TFs, and the dissection of the specific role of each phytohormone through exogenous treatment expermentations are needed for a thorough understanding of the regulation of lemon peel formation and the influence of this process on fruit quality.

## Conclusion

This study unveils the dynamic molecular mechanisms controlling smooth lemon peel formation through combined transcriptomics and metabolomics analyses of developing peels at five stages (C1, C2, C3, C4, and C5). We found that lemon peel development is stage-specifically regulated, highlighting key involved molecular mechanisms, DM, and DEGs. The lemon peel development processes could be divided into three main periods: (1) from 0 DAF to 60 DAF; (2) from 60 DAF (C2) to 90 DAF (C3); and (3) from 90 DAF to physiological maturity. The period between C2 and C3 was the most critical for smooth peel formation. Notably, major metabolic adjustments (down-regulation of flavonoids, terpenoids, phenolic acids, phytohormones, etc.) and transcriptional switches occurred between the transition from C2 to C3. Plant hormone signaling was the most essential enriched pathway during peel development process. Particularly, crosstalks between JA, ABA, SA, GA, and ethylene dynamically and coordinately regulate lemon peel development. Other key potential candidate genes, including TFs and genes that promote green degradation during late stages were identified. Our findings offer an insightful molecular understanding of the genetic control of lemon peel development. Moreover, they represent fundamental resources for the functional dissection of involved regulatory networks.

## Materials and methods

### Plant materials

This study analyzed our previously generated and validated LC-MS (Liquid chromatography-mass spectrometry)-based widely targeted metabolomics and Illumina Hiseq transcriptome data of developing smooth peels of the lemon (*Citrus limon* (L.) Burm) variety “Yunning No.1” at C1 (30 DAF, day after flowering), C2 (60 DAF), C3 (90 DAF), C4 (120 DAF), and C5 (150 DAF) [3].

“Yunning No.1” lemon, a mutant of Eureka lemon, is originated and the main cultivated lemon variety in Yunnan province of China [36]. It is primarily grown in tropical areas of Yunnan province, with sporadic cultivation in Guangxi, Sichuan, Hainan, and Guangdong provinces of China. It produces fruits twice a year: flowering in spring and ripening in autumn (fruits with smooth peels), and flowering in winter and ripening in spring (fruits with rough peels). The average weight, average longitudinal diameter, and average equatorial diameter of a single fruit at the physiological maturity stage are 126.09 g, 81.41 mm, and 57.73 mm, respectively (Fig. 1B-D). The fruit samples with smooth peels analyzed in this study are presented in Fig. 1A. Samples were collected from six-year-old grafted disease- and insect-free plants grown at the Lujiangba Experimental base of the Institute of Tropical and Subtropical Economic Crops, Yunnan Academy of Agricultural Sciences (21°59’N, 98°53’E). The experimental base lies in a subtropical dry-hot climate zone with an average annual sunshine of 2,329.7 h, average radiation of 138,449 cal/(year.cm^2^), average annual temperature of 21.3 °C (10-year average), and an average rainfall of 755.3 mm. The soil quality in the location is sandy loam with medium fertility. Three plants were selected for each time point, and triplicate samples were harvested. The harvested fruits were surface-cleaned with distilled water and wiped, then the peels were removed and wrapped in aluminium foil. Samples were frozen in liquid nitrogen and stored at ‒ 80 °C until metabolomics and transcriptomics analyses.

### Metabolome analysis

100 mg of peel samples were ground with liquid nitrogen and mixed with 80% methanol and 1% formic acid. After 5 min incubation on ice, the mixture was centrifuged at 15,000 g for 20 min at 4 °C. The supernatant was diluted to a final concentration containing 53% methanol by adding LC-MS grade water. The solution was centrifuged again, filtered (0.22 μm micropore membrane filter, SCAA-104), and the resulting supernatant was used for liquid chromatography with tandem mass spectrometry (LC-MS/MS) [37]. The ultra-high performance (UHP) LC-MS/MS analysis was conducted at Novogene Co., Ltd. (Beijing Novogene Technology Co., Ltd) using the QTRAP^®^ 6500 + mass spectrometer and the ExionLC™ AD system (SCIEX, Framingham, MA, USA). The conditions were the following: UPLC: column, Agilent SB-C18 (Santa Clara, CA, USA, 1.8 μm, 2.1 mm × 100 mm); mobile phase was composed of solvent A (pure water with 0.1% formic acid) and solvent B (mixed acetonitrile and 0.1% formic acid). The gradient program was: starting conditions, 95% A, 5% B; within 9 min, a linear gradient to 5% A, 95% B; and a composition of 5% A, 95% B was kept for 1 min; then, a composition of 95% A and 5.0% B was adjusted within 1.1 min and kept for 2.9 min; the flow velocity was set as 0.35 mL/min; the column oven was set to 40 °C; the injection volume was 2 µL; the effluent was alternatively connected to an ESI-triple quadrupole-linear ion trap (QTRAP)-MS. The Electrospray Ionization (ESI) source system was set to: source temperature 500 °C; ion spray voltage (IS) 5500 V (positive ion mode)/−4500 V (negative ion mode). The ion source gas I (GSI), gas II (GSII), and curtain gas (CUR) were maintained at 50, 60, and 25 psi, respectively. The CAD (collision-activated dissociation) was high.

The raw data files obtained were processed using the Compound Discoverer 3.1 (CD3.1; Thermo Fisher Scientific, Waltham, MA, United States) to perform peak alignment, peak picking, and quantitation for each metabolite. Briefly, the data were screened by retention time and mass-to-charge ratio. For an accurate metabolite identification, peak alignment was performed according to retention time deviation and mass deviation (part per million, ppm). Following this, signal-to-noise ratio, adduct ion, and peak area were quantified in ppm. We then identified the metabolites by comparing the quantified data with mzCloud, mzVault, and the MassList primary database search library. Metabolites with a coefficient of variation less than 30% in the QC sample were retained as the final identification result for subsequent analyses [38, 39]. The identified metabolites were annotated using the KEGG, HMDB, and LIPIDMaps databases.

Signal intensities were log-transformed, followed by multivariate analyses in R (vs. 4.3.0). The R packages, including pheatmap, MetaboAnalystR, and prcomp were used for hierarchical clustering analysis (HCA), orthogonal partial least squares discriminant analysis (OPLS-DA), and principal component analysis (PCA), respectively. DMs (differential metabolites) were detected using the ggplot2 program. The selection thresholds were *p*-value < 0.05, |Log2FC| ˃ 1, and VIP ≥ 1. Significant pathways were revealed through KEGG enrichment analysis (http://www.kegg.jp/kegg/pathway.html). Six replications of samples at each developmental stage were analyzed.

### Transcriptome analysis

Three replications of samples at each developmental stage were subjected to total RNA extraction, cDNA library construction, and transcriptome sequencing at Novogene (Beijing NuoheZhiyuan Technology Co., Ltd). Briefly, total RNA was extracted using the Spin Column Plant total RNA Purification Kit (Sangon Biotech, Shanghai, China). The purity of extracted RNAs was assessed on 1% agarose gels and quantified using an Agilent Bioanalyzer 2100 (Agilent Technologies, Sta. Clara, CA, United States). Sequencing libraries were created using the NEB Next Ultra RNA Library Prep Kit following the instructions of the manufacturer. Paired-end cDNA libraries with an insert size of 300 bp were constructed. The clustering of the index-coded samples was performed on a cBot Cluster Generation System using TruSeq PE Cluster Kit v3-cBot-HS (Illumina, San Diego, CA, United States) according to the instructions of the manufacturer. After cluster generation, the library preparations were sequenced on a Novaseq (Illumina, San Diego, CA, United States) platform, and 150 bp paired-end reads were generated. The raw transcriptome data was processed as we previously described [3].

Differentially expressed genes (DEGs) detection was performed with the DESeq2 software [40], with criteria of FDR (false discovery rate) ˂ 0.05 and |Log2fold change| ≥ 1. GO and KEGG pathway enrichment analyses were carried out using Blast2GO [41] and KOBAS2.0 [42] programs, respectively. Significantly enriched terms/pathways were identified at a *p* or q value of ≤ 0.05. In addition, Microsoft Excel was used to organize the data and TBtools was used to generate Venn diagrams and heatmaps [43].

## Supplementary Information

Supplementary Material 1.

Supplementary Material 2.

## Data Availability

All data analyzed in this study are from our previous study [3]. The Transcriptome data have been submitted to NCBI SRA bioproject/accession number: **PRJNA716747**. All other datasets generated and/or analyzed during the current study are available from the corresponding author upon reasonable request.
