# Supplementary material for: Combined metabolome and transcriptome profiling provides insights into dynamic molecular control of lemon (Citrus Limon L.) peel development
Source: BMC Plant Biol. 2025 Nov 25;25:1638. doi: 10.1186/s12870-025-07674-5 (PMC12648855; doi:10.1186/s12870-025-07674-5)
Supplement: Supplementary file 2 — Supplementary Material 2. [file 12870_2025_7674_MOESM2_ESM.pdf]

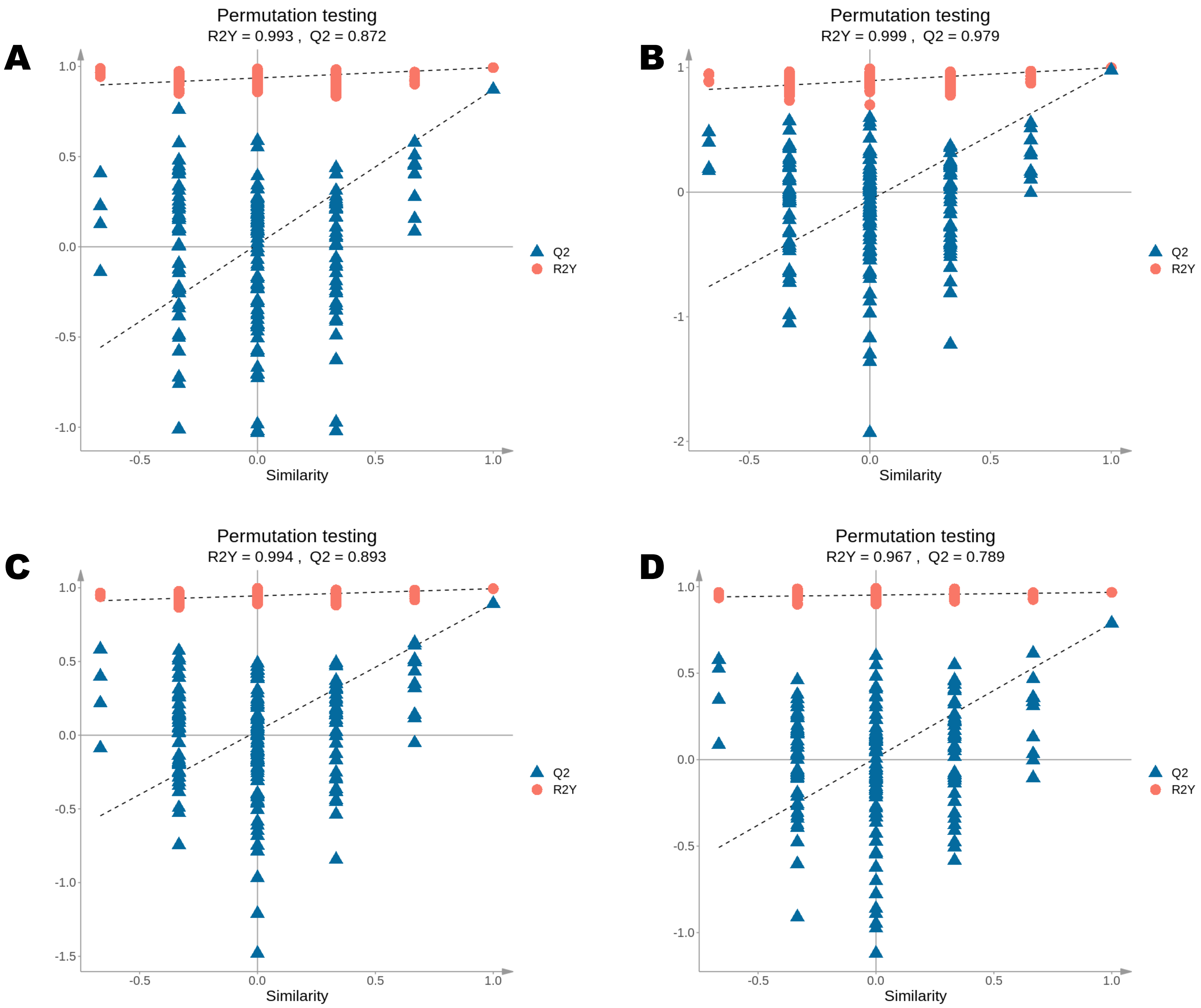

Fig. S1. Permutation plots of OPLS-DA for pairwise comparison between C1.vs.C2 (A), C2.vs.C3 (B), C3.vs.C4 (C), and C4.vs.C5 (D), respectively. C1, C2, C3, C4, and C5 indicate developing lemon peels at 30 DAF (day after flowering), 60 DAF, 90 DAF, 120 DAF, and 150 DAF, respectively.

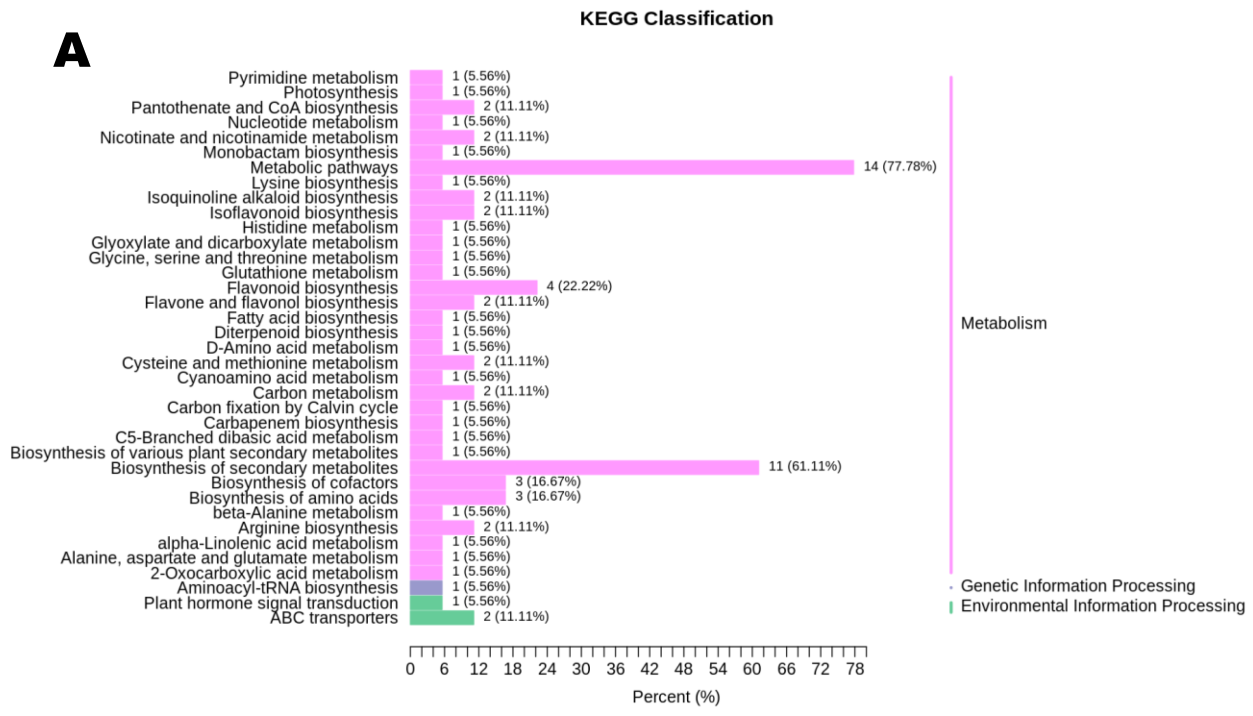

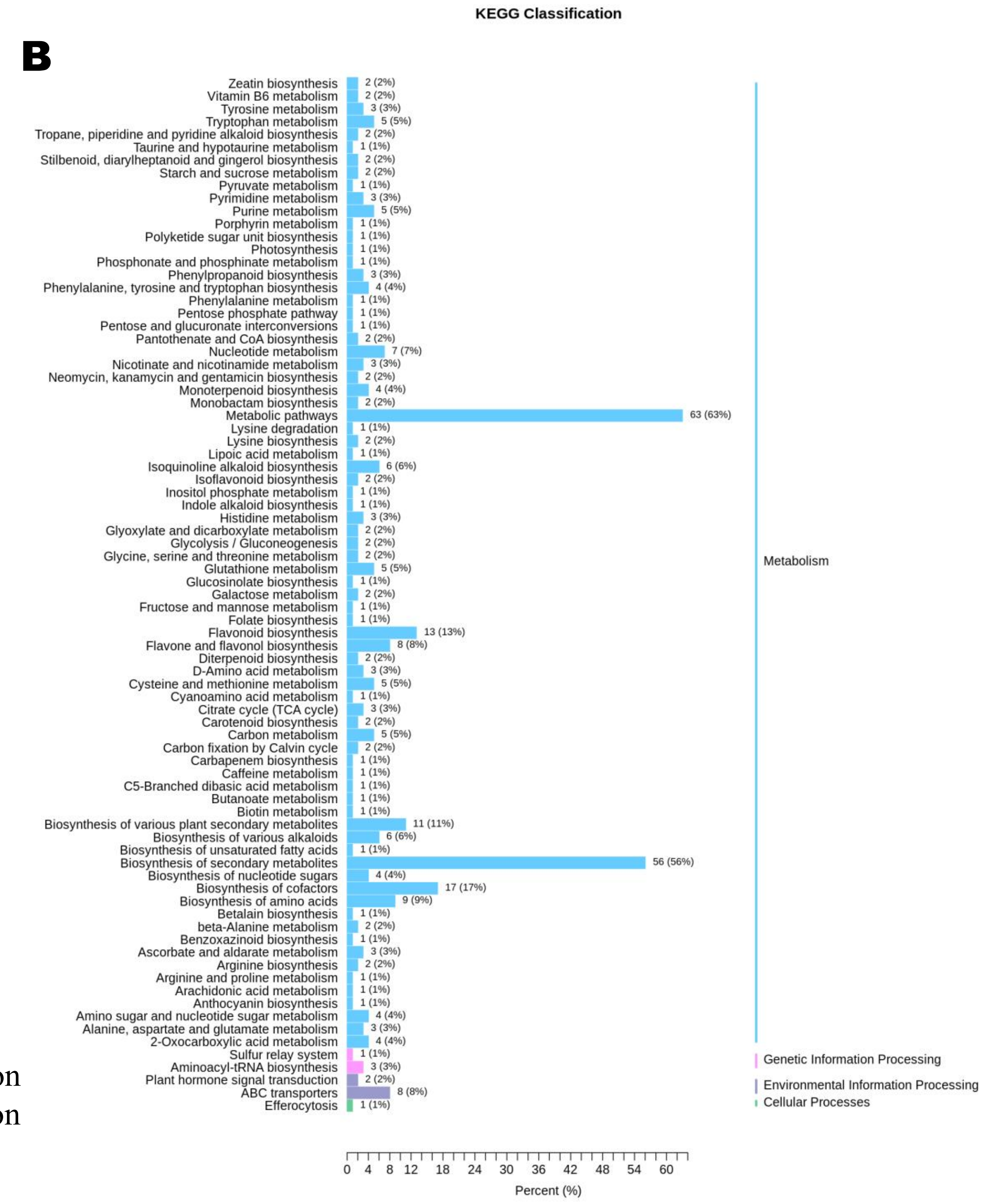

Fig. S2. KEGG annotation results for differential metabolites in pairwise comparison between C1.vs.C2 (A) and C2.vs.C3 (B). C1, C2, and C3 indicate developing lemon peels at 30 DAF (day after flowering), 60 DAF, and 90 DAF, respectively.

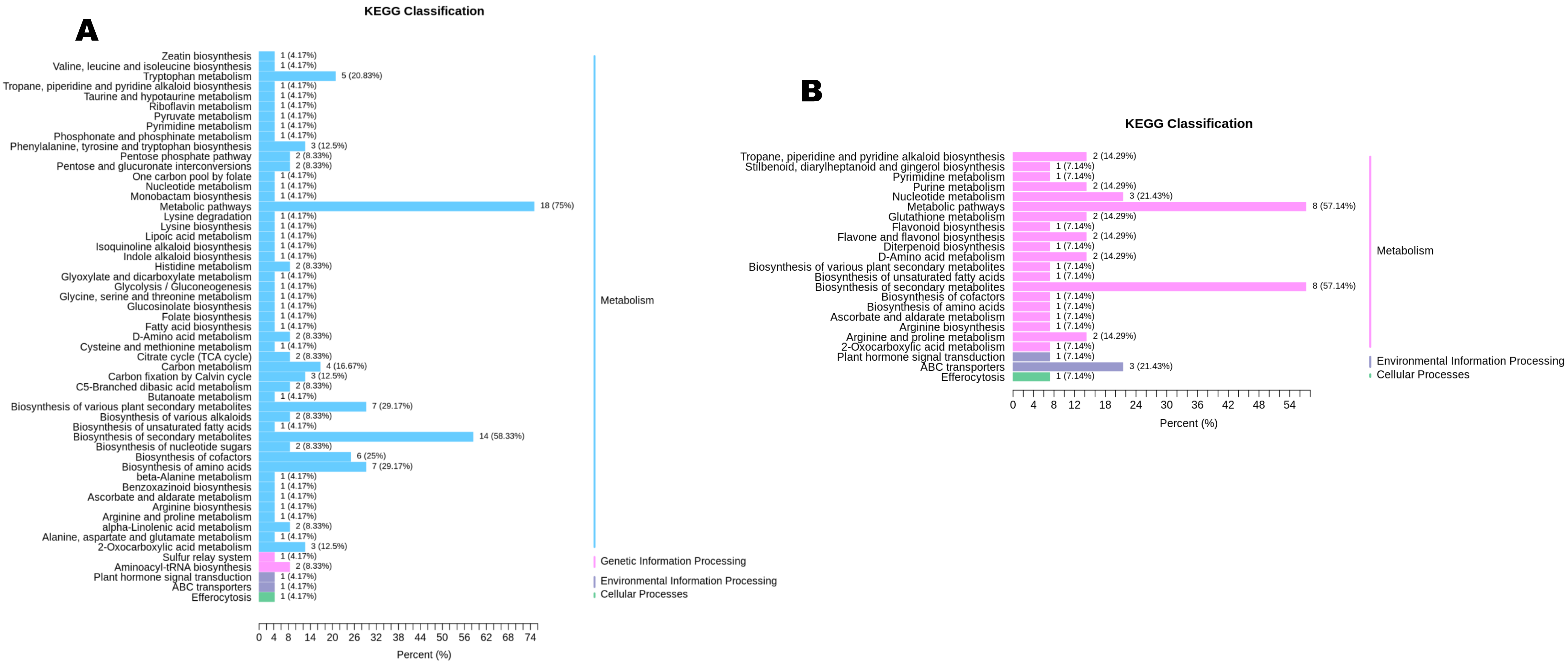

Fig. S3. KEGG annotation results for differential metabolites in pairwise comparison between C3.vs.C4 (A) and C4.vs.C5 (B). C3, C4, and C5 indicate the developing lemon peels at 90 DAF, 120 DAF, and 150 DAF, respectively.

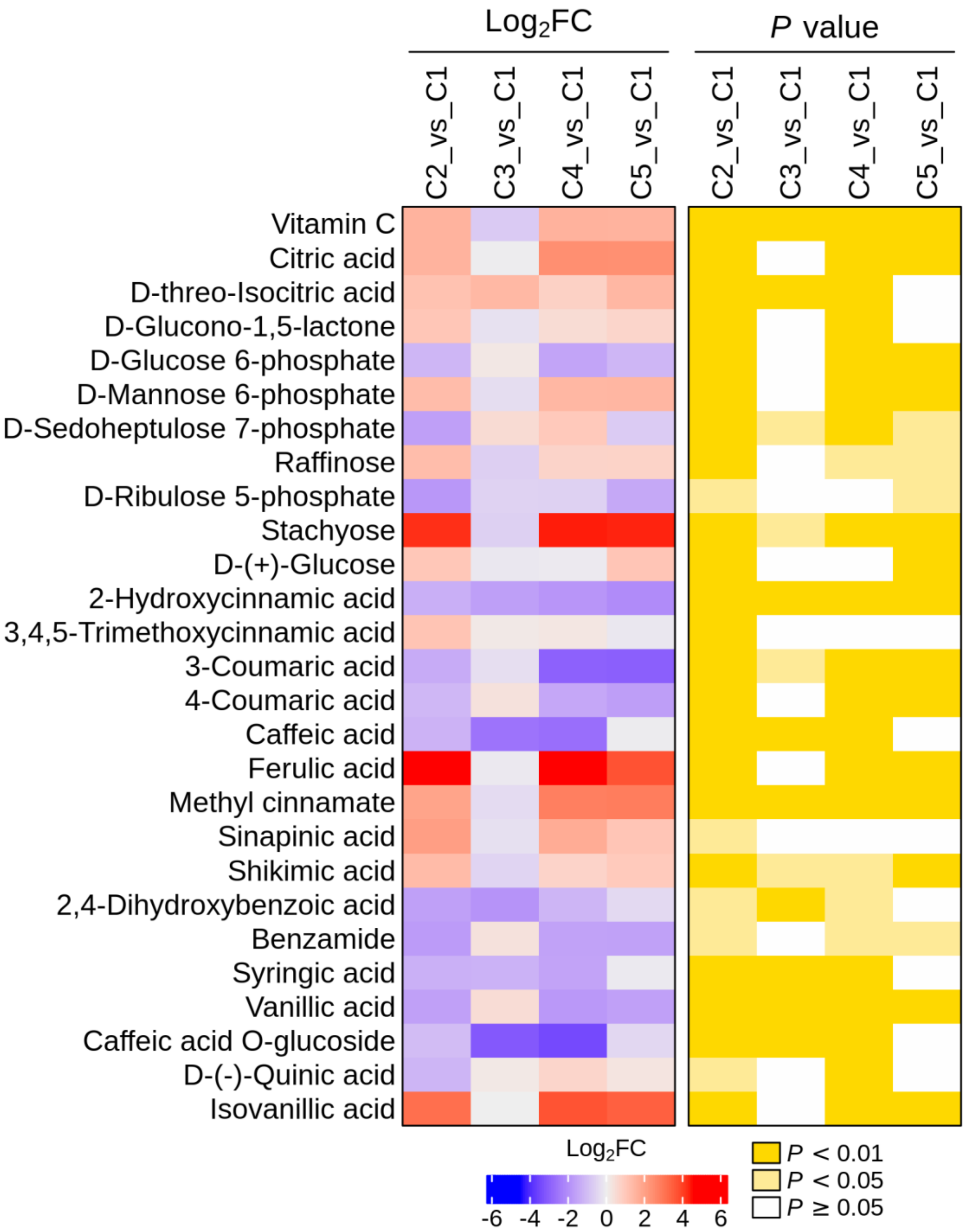

Fig. S4. Accumulation patterns of key differential phenolic acids, organic acids, and carbohydrates in developing lemon peels. C1, C2, C3, C4, and C5 indicate developing lemon peels at 30 DAF (day after flowering), 60 DAF, 90 DAF, 120 DAF, and 150 DAF, respectively.

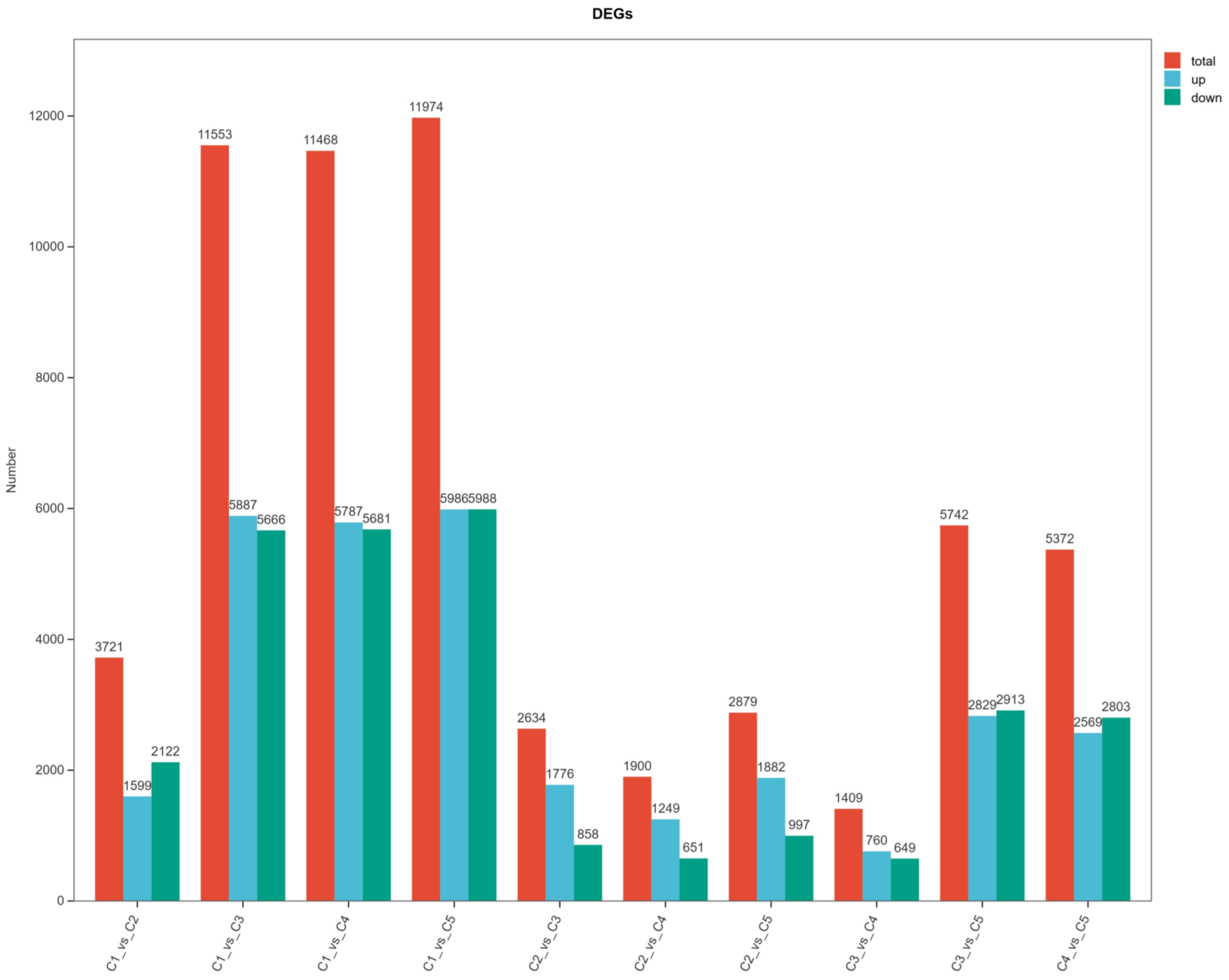

Fig. S5. Number of DEGs in all pairwise comparisons. C1, C2, C3, C4, and C5 indicate developing lemon peels at 30 DAF (day after flowering), 60 DAF, 90 DAF, 120 DAF, and 150 DAF, respectively.

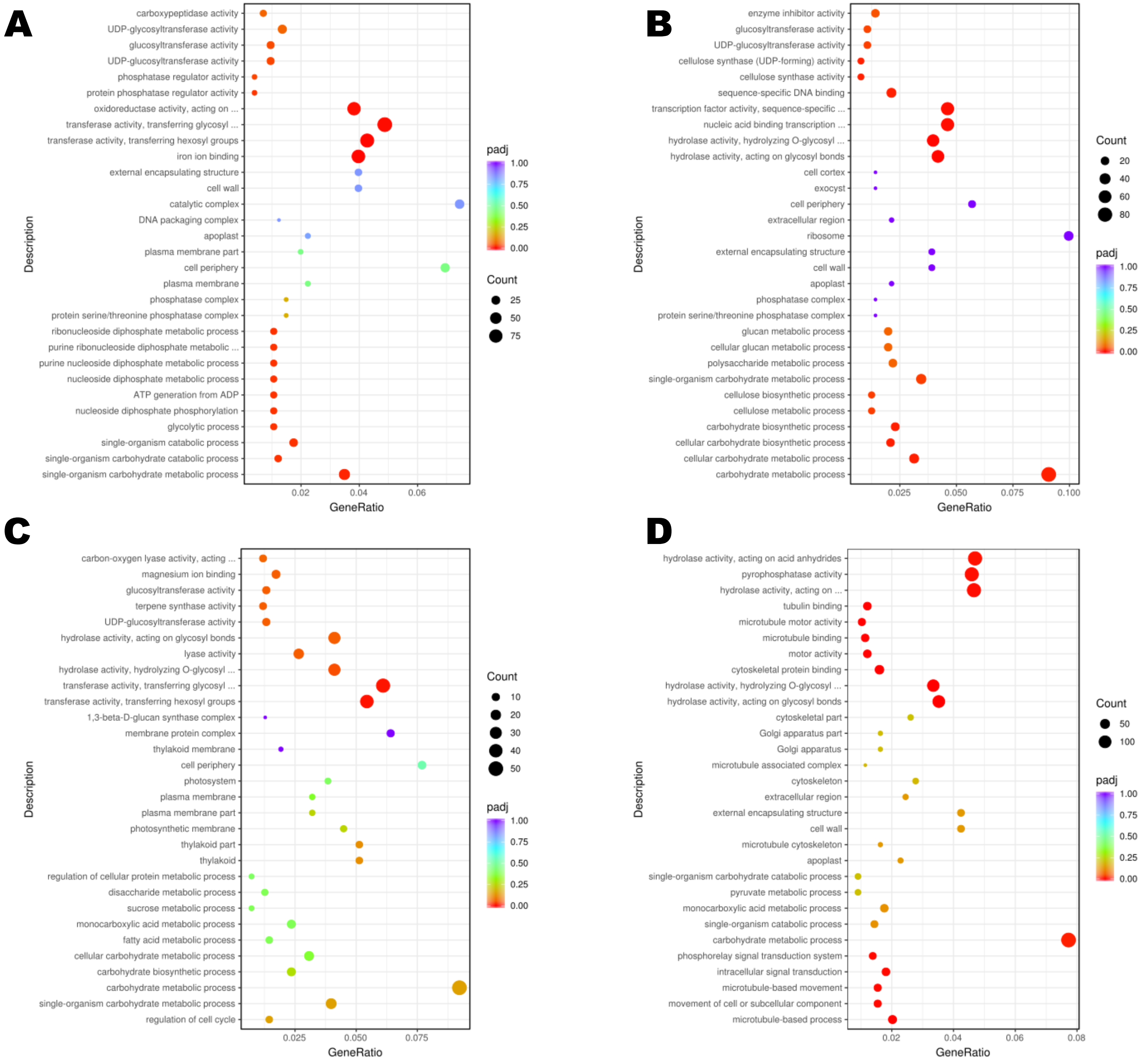

Fig. S6. GO enrichment results for DEGs in pairwise comparison between C1.vs.C2 (A), C2.vs.C3 (B), C3.vs.C4 (C), and C4.vs.C5 (D), respectively. C1, C2, C3, C4, and C5 indicate the developing lemon peels at 30 DAF (day after flowering), 60 DAF, 90 DAF, 120 DAF, and 150 DAF, respectively.
